# Supplementary material for: Prognostic Value of the Lung Immune Prognosis Index Score for Patients Treated with Immune Checkpoint Inhibitors for Advanced or Metastatic Urinary Tract Carcinoma
Source: Cancers (Basel). 2023 Feb 7;15(4):1066. doi: 10.3390/cancers15041066 (PMC9954148; doi:10.3390/cancers15041066)
Supplement: Supplementary file 1 [file cancers-15-01066-s001.zip › cancers-2172200-supplementary.pdf]

Supplementary Figure S1 : OS to LIPI and Bellmunt score in different subgroup

A OS according to Bellmunt score in bladder cancer supbgroup

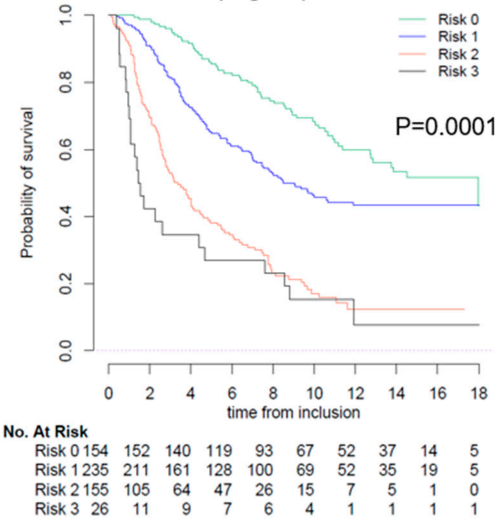

B OS according to LIPI score in bladder cancer subgroup

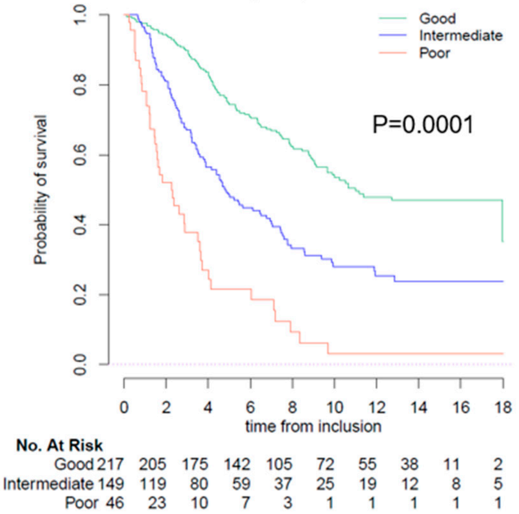

C OS according to Bellmunt score in upper tract cancer subgroup

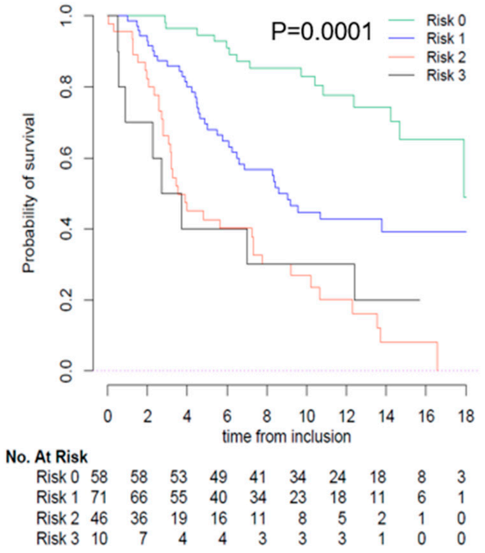

D OS according to LIPÏ score in upper tract cancer subgroup

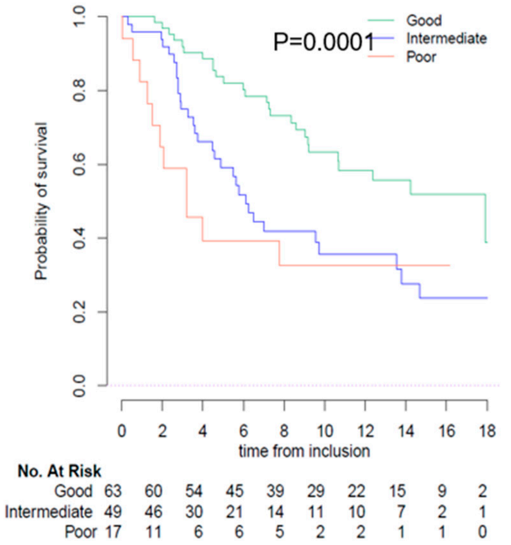

E OS according to Bellmunt score in locally advanced cancer supbgroup

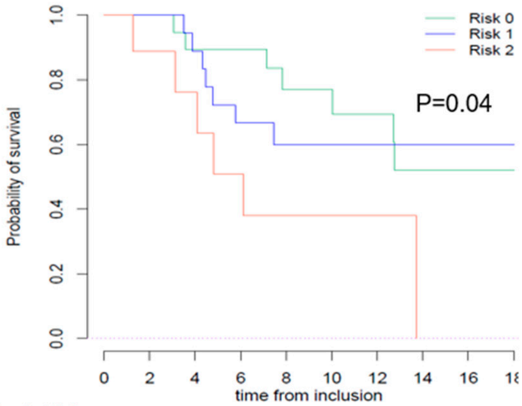

No. At Risk

Risk 0	19	19	17	17	12	10	8	5	1	1
Risk 1	20	19	16	11	9	9	8	6	3	1
Risk 2	9	8	6	4	2	1	1	0	0	0

F OS according to LIPI score in locally advanced cancer subgroup

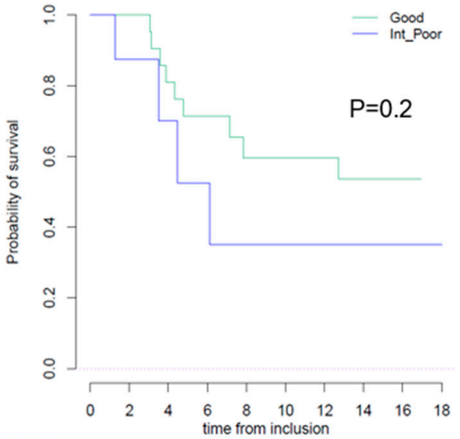

No. At Risk

Good	21	21	17	14	10	10	10	8	2	0
Int_Poor	8	6	4	3	1	1	1	1	1	1

G OS according to Bellmunt score in metastatic cancer subgroup

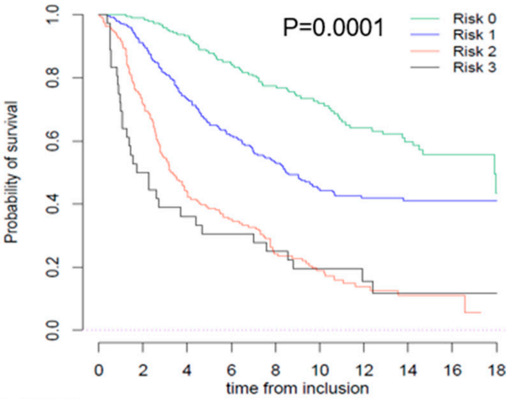

No. At Risk

Risk 0	193	191	176	151	122	91	68	50	21	7
Risk 1	286	258	200	157	125	83	62	40	22	5
Risk 2	192	133	77	59	35	22	11	7	2	0
Risk 3	36	18	13	11	9	7	4	2	1	1

H OS according to LIPÏ score in upper tract cancer subgroup

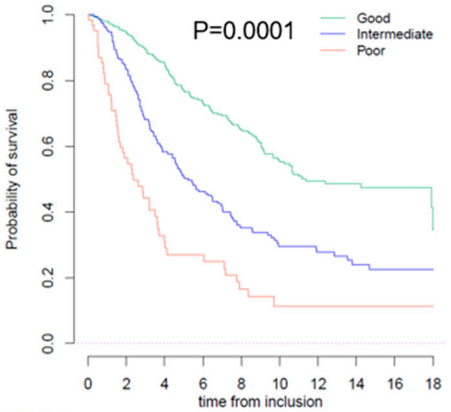

No. At Risk

Good	259	244	212	173	134	91	67	45	18	4
Intermediate	191	159	106	77	50	35	28	18	9	5
Poor	62	34	16	13	8	3	3	2	2	1

Supplementary Table S1: ICI and SAUL cohort characteristics according to LIPI group

	ICI cohort N=137				SAUL cohort N=541			
**Characteristics**	**LIPI Score**							
	**Good N=77**	**Intermediate N=48**	**Poor N=12**	***p-value***	**Good N=280**	**Intermediate N=198**	**Poor N=63**	**p-value**
**Gender** (n, %)<br>Male<br>Female	 66 (86)<br>11 (14)	 37 (77)<br>11 (23)	 9 (75)<br>3 (25)	 *0.37*	 215 (77)<br>65 (23)	 146 (74)<br>52 (26)	 47 (76)<br>16 (24)	 0.738
**Median age at diagnosis** (yrs) (median, range)	69 [63;75]	67.5 [56;74]	66 [60;69]	*0.557*	68 [61;74]	68.5 [60;73]	66 [59.5;74]	0.554
**Smoking history**<br>>10 PYs<br><10 PYs<br>Unknown	 53 (69)<br>20 (26)<br>4 (5)	 34 (71)<br>10 ((21)<br>4 (8)	 8 (67)<br>1 (8)<br>3 (25)	 *0.614*	 195 (70)<br>85 (30)<br>0 (0)	 125 (63)<br>73 (37)<br>0 (0)	 41(65)<br>22 (35)<br>0 (0)	 0.508
**Primary tumor** (n, %)<br>Bladder<br>Upper tract<br>Urethra<br>Other	62 (80)<br>14 (18)<br>1 (2)<br>0 (0)	40 (83)<br>6 (13)<br>2(5)<br>0 (0)	7 (58)<br>5 (42)<br>0 (0)<br>0 (0)	*0.314*	217 (78)<br>54 (19)<br>2 (1)<br>7 (2)	149 (75)<br>43 (22)<br>3 (1.5)<br>3 (1.5)	46 (73)<br>14 (22)<br>0 (0)<br>3 (5)	--
**Histology** (n,%)<br>Pure urothelial or mixed histology<br>Non-urothelial histology<br>-Bellini collecting duct<br>-Glandular neoplasms<br>-Neuroendocrine tumors<br>-Squamous neoplasms	71 (92)<br>0 (0)<br>1 (1)<br>2 (3)<br>3 (4)	45 (94)<br>0 (0)<br>0 (0)<br>0 (0)<br>3 (6)	12 (100)<br>0 (0)<br>0 (0)<br>0 (0)<br>0 (0)	*0.321*	271 (97)<br>3 (1)<br>2 (1)<br>0 (0)<br>2 (1)	187 (94)<br>1 (1)<br>1 (1)<br>4 (2)<br>5 (2)	61 (97)<br>1 (1.5)<br>0 (0)<br>0 (0)<br>1 (1.5)	0.312
**PD-L1 status** (n,%)<br>Positive<br>Negative<br>Unknown	 9 (12)<br>18 (23)<br>50 (65)	 7 (15)<br>14 (29)<br>27 (56)	 2 (17)<br>2 (17)<br>8 (66)	 *0.904*	 80 (29)<br>183 (65)<br>17 (6)	 59 (30)<br>124 (63)<br>15 (7)	 11 (17)<br>44 (70)<br>8 (13)	 0.214
**Type of prior treatment** (all lines combined)<br>Platinum-based therapy<br>Gemcitabine<br>Vinflunine<br>Taxane<br>Other	 58 (75)<br>1<br>3<br>13<br>5	 37 (77)<br>1<br>2<br>15<br>3	 10 (83)<br>0<br>2<br>2<br>0	 --	 276 (99)<br>3<br>0<br>0<br>2	 189 (95)<br>8<br>1<br>0<br>1	 62 (98)<br>1<br>0<br>0<br>0	 --
**Pretreatment performance status (ECOG)** (n,%)<br>0-1<br>≥2<br>Unknown	 62 (81)<br>13 (17)<br>2 (2)	 35 (73)<br>13 (27)<br>0 (0)	 9 (75)<br>3 (25)<br>0 (0)	 *0.372*	 264 (94)<br>16 (6)<br>0 (0)	 180 (91)<br>18 (9)<br>0 (0)	 45 (71)<br>18 (29)<br>0 (0)	 <0.001
**Liver metastatic site** (n,%)	9 (12)	6 (13)	6 (50)	*0.001*	75 (27)	77 (39)	33 (52)	--
**Prior anticancer therapy lines** (median, range)	1 (1;1)	1 (0.75;1)	1 (1;2)	*0.443*	1 (0;1)	1 (0;1)	1 (0;1)	0.048
**Circulating inflammatory markers** (median, range)								

Hemoglobin (g/dL)	12.6 (11.4;13.4)	10.9 (10.1;12.7)	10.8 (9.6;12.2)	*<0.001*	12.2 (11;13.3)	11.2 (10.2;12.5)	10.3 (9.6;11.1)	*<0.001*
Leucocytes (Giga/L)	7.4 (6.0; 9.7)	7.5 (5.8;9.5)	6.8 (5.5;8.6)	*0.917*	6.5 (5.3; 8.1)	8.0 (6.1;10.6)	10 (8.4;13.5)	0.677
ANC (Giga/L)	4.1 (3.2;5.2)	5.6 (4.4;8.2)	8.8 (5.2;11.8)	*<0.001*	4.2 (3.3;5.4)	5.8 (4.2;8.2)	8.6 (6.7;10.7)	*<0.001*
Albumin (g/L)	42 (38.5;44)	39 (36.5;41)	38 (31.5;39.8)	*0.002*	40 (37;43)	38 (34;41)	34 (31;37)	
**LDH > ULN** (n,%)	0 (0)	21 (44)	12 (100)	*<0.001*	0 (0)	106 (54)	63 (100)	<0.001
**dNLR >3** (n,%)	0 (0)	27 (56)	12 (100)	*<0.001*	<0.001	92 (46)	63 (100)	<0.001
**Radiological response**								
Complete response	14 (18)	3 (6)	0 (0)	--	11 (4)	2 (1)	1 (2)	--
Partial response	16 (21)	10 (21)	1 (8)		31 (11)	13 (7)	4 (6)	
Stable disease	18 (23)	7 (15)	1 (8)		115 (41)	54 (27)	6 (9)	
Progressive disease	29 (38)	27 (56)	10 (84)		82 (29)	64 (32)	15 (24)	
Not evaluable	0 (0)	1 (2)	0 (0)		41 (15)	65 (33)	37 (59)	
**Progression-free survival** (mo) (median, range)	5.8 (3.4;17.7)	2.9 (1.7;9.2)	1.3 (1-NR)	*<0.0001*	4.0 (3.1;4.5)	2.2 (2.1;2.4)	1.7 (1.4;2.0)	<0.0001
**Overall survival** (mo) (median, range)	19.7 (13.0;40.7)	13.7 (6.9;25.8)	5.4 (2.5;NR)	*0.002*	12.4 (10.0;NR)	5.4 (4.5;6.9)	2.4 (1.6;3.7)	<0.0001

LIPI : lung immune prognosis index

Supplementary Table S2: Univariate Analysis for OS and PFS in the ICI and SAUL cohorts; Univariate analysis for OS in the Chemo cohort

	**ICI cohort**		**SAUL cohort**		**Chemo cohort**
**Variables**	**HR (95% CI) OS**	**HR (95% CI) PFS**	**HR (95% CI) OS**	**HR (95% CI) PFS**	**HR (95% CI) OS**
**Age at diagnosis** (yrs)<br>≤ 65<br>> 65<br>*P* value	 1 [reference]<br>1.10<br>(0.76;1.60)<br>0.62	 1 [reference]<br>1.32<br>(0.86;2.03)<br>0.20	 1 [reference]<br>0.90<br>(0.80;1.03)<br>0.12	 1 [reference]<br>0.93<br>(0.78;1.10)<br>0.37	 1 [reference]<br>1.20<br>(0.75;1.94)<br>0.45
**Gender**<br>Female<br>Male<br>*P* value	 1 [reference]<br>0.49<br>(0.32;0.75)<br>0.001	 1 [reference]<br>0.67<br>(0.41;1.10)<br>0.11	 1 [reference]<br>0.96<br>(0.83;1.12)<br>0.62	 1 [reference]<br>0.91<br>(0.75;1.10)<br>0.35	 1 [reference]<br>1.59<br>(0.87;2.93)<br>0.14
**Metastatic site**<br>**Liver**<br>No<br>Yes<br>*P* value<br>**Central nervous system**<br>No<br>Yes<br>*P* value	 <br>1 [reference]<br>1.42<br>(0.93;2.18)<br>0.105<br><br>1 [reference]<br>2.76<br>(1.12;6.81)<br>0.028	 <br>1 [reference]<br>1.96<br>(1.23;3.111)<br>0.005<br><br>1 [reference]<br>1.20<br>(0.36;3.81)<br>0.007	 <br>1 [reference]<br>1.80<br>(1.57;2.06)<br><0.0001<br><br>1 [reference]<br>2.12<br>(1.25;3.60)<br>0.005	 <br>1 [reference]<br>2.21<br>(1.86;2.61)<br><0.0001<br>1 [reference]<br>2.72<br>(1.25;4.13)<br>0.007	 <br>1 [reference]<br>0.95<br>(0.56;1.62)<br>0.86<br><br>1 [reference]<br>0.60<br>(0.15;2.47)<br>0.86
**Pretreatment Performance Status (ECOG)**<br>0 to 1<br>≥2<br>*P* value	 1 [reference]<br>1.68<br>(1.30;2.18)<br><0.0001	 1 [reference]<br>3.91<br>(2.43;6.29)<br><0.0001	 1 [reference]<br>2.70<br>(2.19;3.34)<br><0.0001	 1 [reference]<br>3.99<br>(3.16;5.03)<br><0.0001	 1 [reference]<br>2.21<br>(1.35;3.61)<br>0.002
**Pretreatment circulating inflammatory markers**<br>**Albumin**<br>≤ 35g/L<br>> 35g/L<br>*P* value<br><br><br>**Hemoglobin**<br>≤ 10g/dL<br>> 10 g/dL<br>*P* value	 <br>1 [reference]<br>0.62<br>(0.39;0.98)<br>0.04<br><br><br>1 [reference]<br>0.58<br>(0.37;0.97)<br>0.036	 <br>1 [reference]<br>0.56<br>(0.34;0.91)<br>0.02<br><br><br>1 [reference]<br>0.43<br>(0.26;0.71)<br>0.001	 <br>1 [reference]<br>0.67<br>(0.58;0.82)<br><0.0001<br><br><br>1 [reference]<br>0.58<br>(0.48;0.71)<br><0.0001	 <br>1 [reference]<br>0.42<br>(0.34;0.51)<br><0.0001<br><br><br>1 [reference]<br>0.40<br>(0.32;0.51)<br><0.0001	 <br>1 [reference]<br>0.37<br>(0.22;0.63)<br><0.0001<br><br><br>1 [reference]<br>0.82<br>(0.72;0.94)<br>0.003
**Pretreatment LIPI**<br>Good<br><br>Intermediate<br><br>Poor<br>*P* value	 1 [reference]<br>1.56<br>(1.03;2.34)<br>3.86<br>(2.04;7.29)<br>0.0005	 1 [reference]<br>1.55<br>(0.97;2.49)<br>3.08<br>(1.56;6.06)<br>0.007	 1 [reference]<br>1.44<br>(1.19;1.73)<br>2.27<br>(1.72;3.00)<br><0.0001	 1 [reference]<br>2.13<br>(1.67;2.72)<br>4.51<br>(3.24;6.27)<br><0.0001	 1 [reference]<br>1.97<br>(1.12;3.48)<br>3.78<br>(1.53;9.97)<br>0.001

OS: Overall survival, PFS: progression-free survival, LIPI: lung immune prognosis index

Supplementary Table S3: Cross-tabulation analysis of patient number according to the Bellmunt and LIPI scores in the SAUL cohort

LIPI factors (No) / Bellmunt factors (No)	0	1	2
**0**	102	43	2
**1**	125	79	20
**2**	49	63	28
**3**	3	13	13

Blue box: Patients with good prognosis when LIPI and Bellmunt scores were combined

Red box: Patient with poor prognosis when LIPI and Bellmunt scores were combined
